# Lsp family proteins regulate antibiotic biosynthesis in *Lysobacter enzymogenes* OH11

**DOI:** 10.1186/s13568-017-0421-2

**Published:** 2017-06-13

**Authors:** Ruping Wang, Huiyong Xu, Yangyang Zhao, Juan Zhang, Gary Y Yuen, Guoliang Qian, Fengquan Liu

**Affiliations:** 1 Institute of Plant Protection, Jiangsu Academy of Agricultural Sciences, Nanjing, 210014 China; 2 College of Plant Protection, Key Laboratory of Integrated Management of Crop Diseases and Pests, Ministry of Education, Nanjing Agricultural University, Nanjing, 210095 China; 3 Department of Plant Pathology, University of Nebraska-Lincoln, Lincoln, NE 68583-0722 USA

**Keywords:** *Lysobacter*, Antibiotic, Lsp, HSAF, WAP-8294A2, Regulation

## Abstract

Ax21 family proteins have been shown to play regulatory roles in plant- and animal-pathogenic species in the bacterial family Xanthomonadaceae, but the protein have not been investigated previously in the non-pathogenic members of this bacterial family. *Lysobacter enzymogenes*, is a non-pathogenic species known for its capacity as a biocontrol agent of plant pathogens. It is also noted for the production of antimicrobial secondary metabolites, heat stable antifungal factor (HSAF) and WAP-8294A2, that have potential for agricultural and pharmaceutical applications. The species also displays type IV pili-dependent twitching motility and the production of multiple extracellular lytic enzymes as additional biocontrol-related traits. Here, we show that *L. enzymogenes* strain OH11 possesses three genes widely separated in the OH11 genome that code for unique Ax21-like proteins (Lsp). By comparing the wildtype OH11 with mutant strains having a single *lsp* gene or a combination of *lsp* genes deleted, we found that each Lsp protein individually is involved in positive regulation of HSAF and WAP-8294A2 biosynthesis, but the proteins collectively do not exert additive effects in this regulation. None of the Lsp proteins were found to influence twitching motility or the production of three extracellular lytic enzymes. This study is the first to provide evidence linking Ax21-family proteins to antibiotic biosynthesis and, hence, adds new insights into the diversity of regulatory functions of Ax21 family proteins in bacteria.

**Electronic supplementary material:**

The online version of this article (doi:10.1186/s13568-017-0421-2) contains supplementary material, which is available to authorized users.

## Introduction

The Ax21 protein family consists of proteins related to Ax21, which was originally identified in the rice-pathogenic bacterium, *Xanthomonas oryzae* pv. *oryzae* (*Xoo*) and thought to activate XA21-mediated immunity in rice (Lee et al. 2009). This protein (referred to as Ax21_Xoo_ in the present study), however, was recently shown not to be involved in triggering an immune response in rice carrying the XA21 pattern recognition receptor (Bahar et al. 2014). This role was found to be performed, instead, by RaxX (Pruitt et al. 2015). In addition, Ax21_Xoo_ was described originally as being secreted by a type I-secretion system (Lee et al. 2009), but it was shown later to be an outer membrane protein whose secretion is associated with outer membrane vesicles (Bahar et al. 2014), and therefore Ax21_Xoo_ was renamed Omp1X (outer membrane protein 1 in *Xoo*) (Park et al. 2014). Although the role of Ax21_Xoo_ in the pathogenicity of *Xoo* is still unclear, it does function in *Xoo* in regulating cell motility, biofilm formation, and expression of signal transduction proteins (Park et al. 2014). Furthermore, we recently demonstrated that Ax21_Xoc_, which is the homologous protein in *X*. *oryzae* pv. *oryzicola* (*Xoc*), the causal agent of bacterial streak disease on rice, is indispensable for full virulence and biofilm formation by *Xoc* (Qian et al. 2013). Ax21-family proteins also have been studied in *Stenotrophomonas maltophilia*, a human pathogenic species related to *Xoo* and *Xoc.* Two Ax21-homologous proteins (Smlt0378 and Smlt0184) were found to be produced by *S. maltophilia* and their secretion via outer membrane vesicles was confirmed (Devos et al. 2015). One protein, Smlt0378, was shown to act as a cell–cell signal that plays a crucial regulatory role in cell motility and virulence in *S. maltophilia* (Ferrer-Navarro et al. 2013; McCarthy et al. 2011).

All previous studies on the function of Ax21 family proteins were limited to plant or human pathogenic species in the bacterial family Xanthomonadaceae. The existence and functionality of Ax21 family proteins in non-pathogenic bacteria has not been reported. The genus *Lysobacter* is a member of the Xanthomonadaceae that contains non-pathogenic species that have been isolated from various ecological habitats (Christensen and Cook 1978; Hayward et al. 2010). The importance of *Lysobacter* to the agricultural and pharmaceutical industries has been increasingly recognized because members of the genus are recognized as biological control agents against plant pathogens (Kobayashi and Yuen 2007) and as producers of antibiotics with great potential as new antimicrobial pesticides and drugs (Xie et al. 2012). One antibiotic of particular note is heat stable antifungal factor (HSAF), which is a tetramate-containing macrocyclic lactam with broad-spectrum antifungal activity produced by *L. enzymogenes* (Yu et al. 2007; Li et al. 2008). This antibiotic is unique in its mode of action, disrupting the biosynthesis of membrane sphingolipids, and it also has a distinctive biosynthetic mechanism (Li et al. 2006, 2014; Lou et al. 2011). Another antibiotic produced by *L. enzymogenes* is WAP-8294A2, a cyclic lipodepsipeptide antibiotic that has activity against Gram-positive bacteria (Kato et al. 1997; Zhang et al. 2011). Because WAP-8294A2 exhibits high anti-MRSA (methicillin-resistant *Staphylococcus aureus*) activity, it is particularly important in medical therapy (Kato et al. 1997; Zhang et al. 2011). In addition to these secondary metabolites, *L. enzymogenes* also produces multiple extracellular lytic enzymes that degrade fungal cell walls components and play important roles in the biological control activity of *L. enzymogenes* (Zhang and Yuen 2000; Palumbo et al. 2003). Furthermore, *L. enzymogenes* produces type IV pili (T4P), which are critical to twitching motility (movement on solid surfaces) and attachment to substrates such as fungal hyphae (Kobayashi et al. 2005; Patel et al. 2011, 2013).

Knowledge of how these traits in *L. enzymogenes* are regulated individually or collectively could facilitate the application of strains for pharmaceutical and biological control purposes, and recent research has begun shed light on this topic. We previously showed that a small fatty acid *Le*DSF3 functions as a cell–cell signal to regulate HSAF biosynthesis via the RpfC/RpfG two-component transduction system and a global transcriptional regulator Clp in *L. enzymogenes* (Qian et al. 2013; Wang et al. 2014; Han et al. 2015). The regulator Clp also controls the production of several extracellular enzymes and twitching motility (Wang et al. 2014). Furthermore, the solo LuxR regulator, LesR, and the response regulator, PilG, were both identified as key factors in modulating HSAF biosynthesis, while only PilG was also involved in regulating twitching motility (Qian et al. 2014; Zhou et al. 2015).

Despite such progress, however, our understanding of the mechanisms regulating antibiotic, lytic enzyme and T4P production in *L. enzymogenes* is still incomplete. Given that Ax21 family proteins produced by plant and animal-pathogenic members of the Xanthomonadaceae have been implicated in cell–cell signaling and regulation of cell motility and other virulence-related traits, we conducted this study to determine whether the non-pathogenic bacterium *L. enzymogenes* produces a Ax21 family protein, and if so, what roles the protein plays in the regulation of antibiotics and lytic enzyme production and motility. In the present work, we found that *L. enzymogenes* strain OH11 possesses three Ax21_Xoo_-like proteins. Because these molecules were small proteins unique to *L. enzymogenes*, we designated them as Lsp1, Lsp2, and Lsp3. All three were found to regulate the biosynthesis of the antibiotic HSAF and WAP-8294A2, but none were involved in regulating T4P-dependent twitching motility and extracellular lytic enzyme production.

## Materials and methods

### Bacterial strains, plasmids and growth conditions

The bacterial strains and plasmids used in this study are listed in Table 1. *Escherichia coli* strain DH5α was used for vector construction and grown in LB broth at 37 °C. Unless otherwise stated, the wild-type OH11 strain of *L. enzymogenes* and its derivatives were grown in LB medium at 28 °C. When required, kanamycin (Km) and gentamicin (Gm) were added to final concentrations ranging from 25 to 150 μg/mL.

**Table 1:** Bacterial strains and plasmids used in this study

Strains and plasmids	Characteristics^a^	Source or citation
*Lysobacter enzymogenes*		
OH11	Wild type, Km^R^	Qian et al. (2009) CGMCC No. 1978
Δ*lsp1*	*lsp1* in-frame deletion mutant, Km^R^	This study
Δ*lsp2*	*lsp2* in-frame deletion mutant, Km^R^	This study
Δ*lsp3*	*lsp3* in-frame deletion mutant, Km^R^	This study
Δ*lsp1*(*lsp1*)	∆*lsp1* harboring plasmid pBBR-*lsp1*, Gm^R^, Km^R^	This study
Δ*lsp2*(*lsp2*)	∆*lsp2* harboring plasmid pBBR-*lsp2*, Gm^R^, Km^R^	This study
Δ*lsp3*(*lsp3*)	∆*lsp3* harboring plasmid pBBR-*lsp3*, Gm^R^, Km^R^	This study
Δ*lsp1*(pBBR)	∆*lsp1* harboring plasmid pBBR1-MCS5, Gm^R^, Km^R^	This study
Δ*lsp2*(pBBR)	∆*lsp2* harboring plasmid pBBR1-MCS5, Gm^R^, Km^R^	This study
Δ*lsp3*(pBBR)	∆*lsp3* harboring plasmid pBBR1-MCS5, Gm^R^, Km^R^	This study
Δ*lsp12*	The *lsp1* & *lsp2* deletion mutant, Km^R^	This study
Δ*lsp23*	The *lsp2*& *lsp3* deletion mutant, Km^R^	This study
Δ*lsp13*	The *lsp1*& *lsp3* deletion mutant, Km^R^	This study
Δ*lsp123*	The triple *lsp* deletion mutant, Km^R^	This study
*Escherichia coli*		
DH5α	F^−^, φ80d*lacZ*ΔM15, ∆(*lacZYA*-*argF*) U169, *deoR*, *recA1*, *endA1*, *hsdR*17(r_k_^−^,m_k_^+^), *phoA*, *supE*44, λ^−^, *thi*-1, *gyrA*96	Qian et al. (2012)
Plasmids		
pEX18GM	Suicide vector with a *sacB* gene, Gm^R^	Hoang et al. (1998)
pBBR1-MCS5	Broad-host-range vector with a P_*lac*_ promoter	Kovach et al. (1995)
pEX18-*lsp1*	pEX18GM with two flanking fragments of *lsp1*, Gm^R^	This study
pEX18-*lsp2*	pEX18GM with two flanking fragments of *lsp2*, Gm^R^	This study
pEX18-*lsp3*	pEX18GM with two flanking fragments of *lsp3*, Gm^R^	This study
pBBR-*lsp1*	pBBR1-MCS5 cloned with a 1275-bp fragment containing intact *lsp1* and its predicted promoter	This study
pBBR-*lsp2*	pBBR1-MCS5 cloned with a 1591-bp fragment containing intact *lsp2* and its predicted promoter	This study
pBBR-*lsp3*	pBBR1-MCS5 cloned with a 1655-bp fragment containing intact *lsp3* and its predicted promoter	This study

^a^Km^R^, Gm^R^ = Kanamycin-, Gentamicin-, respectively

### Mutant generation and complementation

Wild-type OH11 was used as the parent strain for generation of gene in-frame deletion mutants as described previously (Qian et al. 2012). The primers used in this procedure are listed in Table 2. In brief, two pairs of primers were used to amplify an upstream and downstream homologue arm of each *lsp* gene by polymerase chain reaction (PCR). The two homologue arms corresponding to each *lsp* gene were cloned into the appropriate sites of the suicide vector pEX18GM, resulting in the final construct (Table 1). This final construct was transformed into wild-type cells by electrotransformation, as described previously (Han et al. 2015). Transformants were selected on LB plates in the presence of Km (100 μg/mL) and Gm (150 μg/mL) but without sucrose. The positive colonies were selected and further plated on LB plates supplemented with 10% (weight/volume) sucrose and Km (100 μg/mL) to screen for resolution of the construct by a second cross-over event. The resultant mutants were validated by a PCR approach using the respective primers (Table 2). To generate a mutant with two deleted *lsp* genes (i.e., Δ*lsp12,* Δ*lsp23,* or Δ*lsp13*), the suicide vector pEX18GM containing one of the *lsp* genes was introduced into a mutant strain with a different *lsp* gene already deleted. Similarly, the triple mutant Δ*lsp123* was generated by introduction of pEX18GM-*lsp2* into Δ*lsp13*. All the single, double and triple mutants generated in this study were verified by PCR (Additional file 1: Tables S1, S2). For complementation, the DNA fragment containing each full-length *lsp* gene and its native promoter region was amplified from strain OH11 by PCR with the respective primer pairs (Table 2). Each DNA fragment was cloned into the broad-host vector pBBR1-MCS5 (Table 1), and which was then introduced into the corresponding *lsp* mutant, resulting in generation of the plasmid-based complemented strain.

**Table 2:** Primers used for mutant construction and complementation in this study

Primer	Sequence^a^	Purpose
*lsp1*-F1	GGGGTACCACGCCCTCGCACGCCTTCGC (*Kpn*I)	To amplify a 1072-bp upstream homologue arm of *lsp1*
*lsp1*-R1	CCCAAGCTTCATCACCGCCGACGCCAAGT (*Hin*dIII)	
*lsp1*-F2	CCCAAGCTTCGGCGAGGGTGAGGGCAAGC (*Hin*dIII)	To amplify a 638-bp downstream homologue arm of *lsp1*
*lsp1*-R2	GCTCTAGACGGCAGCGGATAGCGGAAGT (*Xba*I)	
*lsp2*-F1	GGGGTACCTTCTGCCCGTGCTGCCTGTT (*Kpn*I)	To amplify a 788-bp upstream homologue arm of *lsp2*
*lsp2*-R1	CCCAAGCTTCTGGGGCATCGTCGCTGAAG (*Hin*dIII)	
*lsp2*-F2	CCCAAGCTTTCGTAGGCGGCGGCGGAGGT (*Hin*dIII)	To amplify a 875-bp downstream homologue arm of *lsp2*
*lsp2*-R2	GCTCTAGACTGCTGTGGGTCGGCGTGCT (*Xba*I)	
*lsp3*-F1	GGGGTACCTCGCTGGAGTGGGGAGGCAT (*Kpn*I)	To amplify a 553-bp upstream homologue arm of *lsp3*
*lsp3*-R1	CCCAAGCTTGCGTCGGTGTGCTGCTCAAG (*Hin*dIII)	
*lsp3*-F2	CCCAAGCTTCGGCTTCCAGGTAGGTGTAG (*Hin*dIII)	To amplify a 379-bp downstream homologue arm of *lsp3*
*lsp3*-R2	GCTCTAGAGCGGCAACTCCAACAAGACC (*Xba*I)	
*lsp1*-F	GGGGTACCGGTCTCGGCTCGCATCGGTC (*Kpn*I)	To amplify a 1275-bp fragment containing intact *lsp1* and its predicted promoter
*lsp1*-R	CCCAAGCTTGCGAGCAGAAGCCCGAGTAC (*Hin*dIII)	
*lsp2*-F	CCCAAGCTTCAAGAAGACCGCCAAGACCG (*Hin*dIII)	To amplify a 1591-bp fragment containing intact *lsp2* and its predicted promoter
*lsp2*-R	GCTCTAGAGACCACCAGGACGGCGAATG (*Xba*I)	
*lsp3*-F	GCTCTAGAAGAATGTCGGCGTCGTCGTC (*Xba*I)	To amplify a 1247-bp fragment containing intact *lsp3*
*lsp3*-R	GGGGTACCTGGGGCGATGGAAGCAAGGG (*Kpn*I)	
Gm-F	GTTAGGTGGCGGTACTTGGGTCG	To amplify a 500-bp DNA fragment of the gentamycin-resistance gene
Gm-R	ATGTTACGCAGCAGCAACGATGT	

^a^Restriction enzyme digestion site are underlined

### Observation of twitching motility

Strain OH11 and its derivatives were examined for twitching motility as described previously (Wang et al. 2014; Zhou et al. 2015). In brief, a thin layer of an agar medium (1/20 TSA containing 1.8% agar) was applied to a sterilized microscope slide and allowed to harden. The edge of a sterilized coverslip was dipped into a cell suspension of the bacterial strain and then gently pressed onto the surface of the agar, creating a thin inoculation line. After 24 h incubation, the bacterial culture on the microscope slide was observed under a compound microscope at 640× magnification without a coverslip. Twitching motility was evident in the form of individual bacterial cells or small clusters of cells at the colony margin growing away from the main colony (Wang et al. 2014; Reichenbach 2006). Three replicate slides for each bacterial strain treatment were examined, and the experiment was performed three times.

### Assay of extracellular lytic enzymes

Production of extracellular chitinase, cellulase and protease by wildtype OH11 and its derivatives was evaluated following methods used previously in our laboratory (Qian et al. 2013, 2014). Briefly, chitin and cellulose hydrolysis were tested using agar media containing 1% colloidal chitin and 1% carboxymethyl cellulose (CMC), respectively (Kobayashi et al. 2005), while proteolytic activity was measured as diffusion-clearing zones on milk agar (Folman et al. 2003). For each experiment involving an enzyme type, there were three replicates assays for each bacterial strains, and the experiment was performed three times.

### HSAF analysis

The wild-type OH11 and its derivatives were evaluated for HSAF production by first cultivating each strain in 1/10 TSB at 28 °C for 13 h (wildtype) or 17 h (mutants); this yielded cultures at early stationary phase and similar population levels as determined from the individual growth curves (Additional file 1: Figure S1). HSAF was extracted from these cultures and detected by HPLC (High-Performance Liquid Chromatography) as described previously (Qian et al. 2013, 2014; Yu et al. 2007). HSAF content was expressed as a ‘peak area/OD_600_’ index as described previously (Wang et al. 2014), where the ‘peak area’ is the area under the peak corresponding to HSAF in the HPLC analysis, while the OD_600_ represents the optical density of the culture at the time point used for HSAF extraction. Each strain was evaluated for HSAF production in three biological experiments, and in each experiment, three replicate cultures of each bacterial strain were assayed.

### Extraction and analysis of WAP-8294A2

The wild-type OH11 and the *lsp* mutants were grown in 1/10 TSB for 13 and 17 h, respectively, and a 1 mL aliquot of each culture was transferred to 50 mL of fresh 1/10 TSB broth, which then was incubate at 28 °C for 3 days with shaking at 200 rpm. The extraction of WAP-8294A2 from broth and HPLC analysis of the antibiotic was performed as described previously (Zhang et al. 2011, 2014). Prior to WAP-8294A2 extraction, 50 mL of culture was centrifuged, and the supernatant was collected, which was further adjusted to a pH of 2.5 with 1 N HCl. Then the acidic supernatant was extracted with n-butanol/ethyl acetate (1/1, vol) containing 0.05% TFA (trifluoroacetic acid). The organic phase was dried, and the residue was dissolved in 2 mL methanol containing 0.05% TFA. This final solution, defined as WAP-8294A2 extract, was used for HPLC analysis.

### Statistical analysis

All analyses were conducted using SPSS 14.0 (SPSS Inc., Chicago, IL, USA). The hypothesis test of percentages (*t* test, *P* = 0.05 or 0.01) was used to identify significant differences in HSAF production between the wild-type OH11 and particular derivative strains.

## Results

### *L. enzymogenes* produces three Ax21_Xoo_-like proteins

**Fig. 1:**
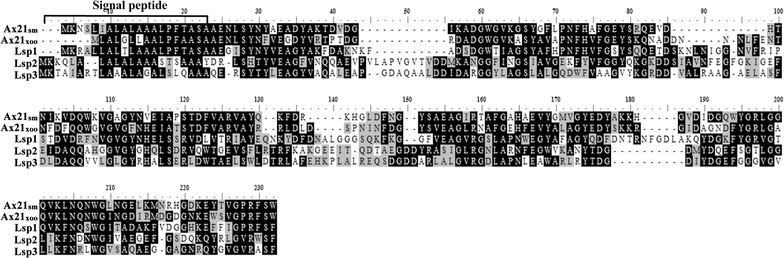
Multiple sequence alignment of Lsp proteins and its homologous proteins. Ax21_Sm_ is Smlt0387 from *Stenotrophomonas maltophilia*; Ax21_Xoo_ is PXO_03968 from *Xanthomonas oryzae* pv. *oryzae*; Lsp1 (KR258786), Lsp2 (KR258787) and Lsp3 (KR258788) are the Ax21_Xoo_ homologous proteins identified from *Lysobacter enzymogenes*. The secretory signal-peptide sequence was indicated, which has been characterized as ‘MKTSLLALGLLAALPFAASA’ from Ax21_Xoo_ (Bahar et al. 2014)

To determine whether *L. enzymogenes* produces Ax21-family proteins, we searched the genome of *L. enzymogenes* strain OH11 for genes coding for proteins homologous to Ax21_Xoo_. This analysis led to identification of three predicted Ax21_Xoo_-homologous proteins in *L. enzymogenes*. Due to they being small molecule weight proteins in *L. enzymogenes*, they were designated as Lsp1 (216 aa), Lsp2 (217 aa) and Lsp3 (216 aa), and they shared 41% (E value 3e−038), 28% (E value 2e−013) and 23% (E value 3e−006) identity, respectively, to Ax21_Xoo_ (194 aa) at the amino-acid level. Furthermore, the Lsp proteins were similar, but not identical, to each other as to amino acid sequence, with Lsp1 sharing 33% (E value 8e−025) and 35% (E value 3e−023) identity to Lsp2 and Lsp3, respectively, and Lsp2 having 43% identity (E value 1e−049) with Lsp3. All three Lsp proteins contained a secretory signal-peptide sequence, similar to that of Ax21_Sm_ and Ax21_Xoo_ (Fig. 1), suggesting that Lsp are secreted proteins. Analysis of the spatial positions of the genes for these proteins revealed them to be separated from each other in the genome of *L. enzymogenes* OH11 (Additional file 1: Figure S2). Collectively, these results indicated that *L. enzymogenes* produces three unique Ax21_Xoo_-like proteins.

### Lsp proteins are not involved in regulating T4P-mediated twitching motility and extracellular lytic enzymes production

**Fig. 2:**
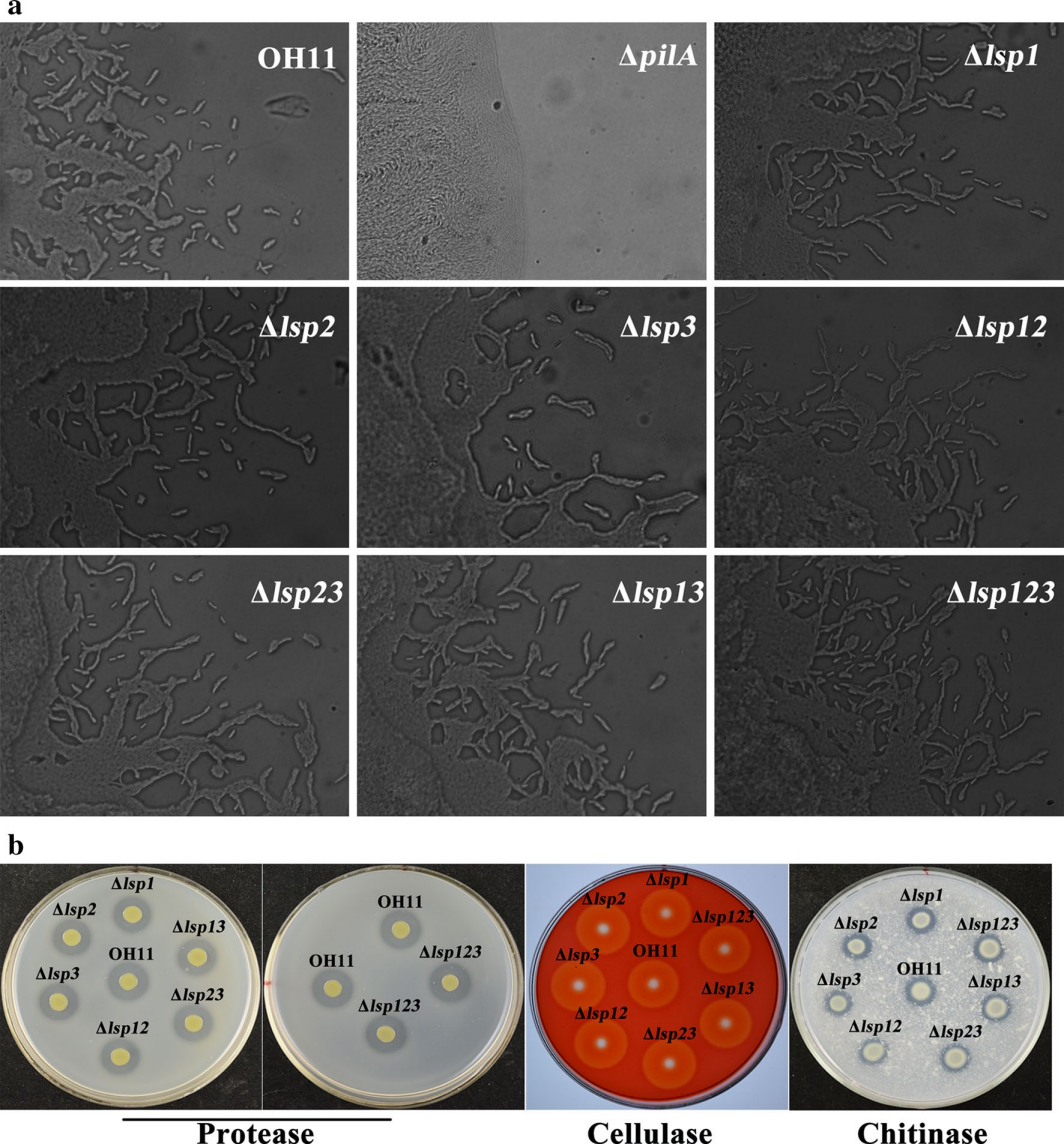
Deletion of Lsp protein genes individually or in various combinations in *Lysobacter enzymogenes* did not disrupt twitching motility (**a**) and production of extracellular protease, cellulase and chitinase (**b**). In **a**, individual cells or cell clusters produced at the margin of colony are characteristic of twitching motility in *L. enzymogenes*. OH11: wild-type strain of *L. enzymogenes*; Δ*pilA*: twitching-motility deficient mutant used as a negative control; Δ*lsp1*, Δ*lsp2* and Δ*lsp3*: mutants with in-frame deletions *lsp1*, *lsp2* and *lsp3*, respectively; Δ*lsp12*, Δ*lsp13* and Δ*lsp23*: double in-frame deletion mutants with double deletions at *lsp1* and *lsp2*, *lsp1* and *lsp3*, and *lsp2* and *lsp3*, respectively; Δ*lsp123*: the triple deletion mutant lacking *lsp1*, *lsp2* and *lsp3*

To explore the function of the Lsp proteins, we generated seven in-frame deletion mutants in strain OH11 (Additional file 1: Tables S1, S2), including three having a single mutation (Δ*lsp1*, Δ*lsp2* and Δ*lsp3*), three containing double mutations (Δ*lsp12*, Δ*lsp13* and Δ*lsp23*), and one triple mutation (Δ*lsp123*). The mutants were evaluated in comparison to the wild-type OH11 for twitching motility and production of extracellular lytic enzymes. When cultured under conditions favorable to expression of twitching motility, we found that all *lsp* mutants, similar to the wild-type OH11, produced mobile cells or cell clusters at the margin of the respective colonies (Fig. 2a), which is indicative of twitching motility in *L. enzymogenes*. This result suggests that the Lsp proteins are not involve in the regulation of T4P-mediated twitching motility in *L. enzymogenes*. When cultured on indicator media for protease, cellulase and chitinase production, all *lsp* mutants displayed wild-type levels in producing these three extracellular lytic enzymes (Fig. 2b), suggesting that Lsp proteins are also not involved in regulating the production of extracellular lytic enzymes by *L. enzymogenes*.

### Lsp proteins play a key role in regulating HSAF biosynthesis

**Fig. 3:**
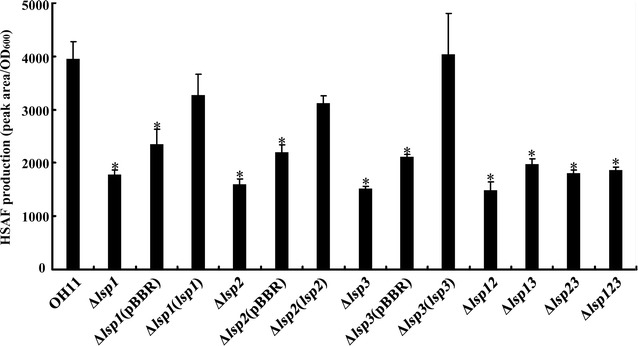
Deletion of *lsp* genes individually or in various combinations in *Lysobacter enzymogenes* significantly impaired HSAF production. The identities of strains OH11, Δ*lsp1*, Δ*lsp2* Δ*lsp3*, Δ*lsp12*, Δ*lsp13*, Δ*lsp23* and Δ*lsp123* are provided in the Fig. 2 caption. Δ*lsp1*(pBBR), Δ*lsp2*(pBBR), and Δ*lsp3*(pBBR) are the respective mutant strain containing the empty vector, pBBR1-MCS5. Δ*lsp1*(*lsp1*), Δ*lsp2*(*lsp2*), Δ*lsp3*(*lsp3*) are the complemented strain of each mutant in which the deleted *lsp* gene(s) was expressed in the plasmid. The values shown in this figure represent the means of three experiments. *Vertical bars* represent standard errors. The *asterisk above a bar* indicates a significant difference (*p* < 0.05) between the respective strain and the wild-type strain OH11

Comparison of wildtype OH11 and its derivative strains revealed the Lsp proteins have a regulatory role in HSAF biosynthesis in *L. enzymogenes* focused on HPLC analysis of the antibiotic extracted from broth cultures showed that a deletion of any one of the *lsp*-genes caused a significant decrease in HSAF production in the mutant strain compared to the wild-type OH11 (Fig. 3). Furthermore, *in trans* complementation of each mutation by introduction of a plasmid containing the intact deleted gene rescued the deficiency of each mutation, whereas introduction of the empty vector into each mutant had no significant effect (Fig. 3). HSAF biosynthesis in each of the three double deletion mutants (Δ*lsp12*, Δ*lsp13* and Δ*lsp23*) and the triple deletion mutant (Δ*lsp123*) also was depressed compared to that of the wild-type, but not entirely eliminated; the decrease in HSAF yield in each double or triple deletion mutant occurred to the similar degree as every single deletion mutant (Fig. 3). These results indicate that each Lsp protein is involved in regulating HSAF biosynthesis in *L. enzymogenes*, but the proteins in dual combinations or all together do not exert additive effects.

### Lsp proteins are involved in the regulation of WAP-8294A2 biosynthesis

**Fig. 4:**
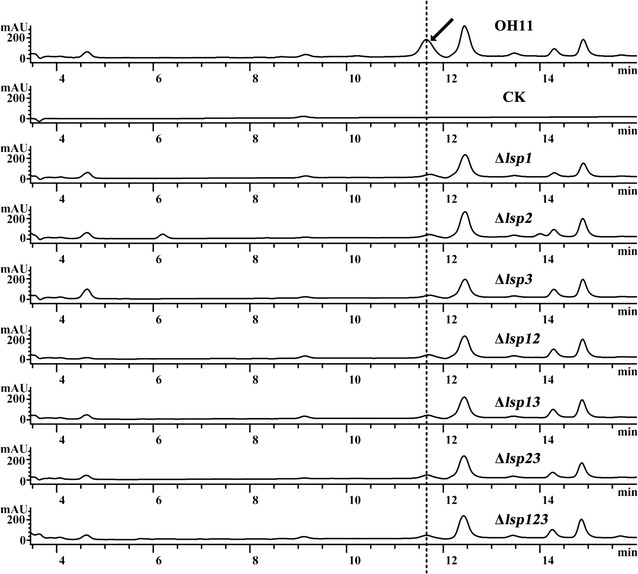
Inactivation of *lsp* genes individually or in various combinations caused a decrease in the WAP-8294A2 production. The identity of each strain in this figure is provided in the captions for Figs. 2 and 3. ‘CK’ is the solvent (methanol)-only control. The *arrow* indicates the peak corresponding to WAP-8294A2

We used the battery of *lsp* mutants to explore the role of Lsp proteins in the regulation of WAP-8294A2 biosynthesis. As shown in Fig. 4, individual deletion of *lsp1*, *lsp2* or *lsp3* led to a visible reduction in WAP-8294A2 yield, suggesting that each Lsp protein played a key role in regulating WAP-8294A2 biosynthesis. Furthermore, the three double mutants (Δ*lsp12*, Δ*lsp13* and Δ*lsp23*), and the triple mutant (Δ*lsp123*) produced WAP-8294A2 at levels similar to that produced by each single mutant, implying that the Lsp proteins do not exert additive effects in regulating WAP-8294A2 biosynthesis.

## Discussion

The involvement of Ax21 family proteins in regulating virulence-related traits was previously documented in certain pathogenic species of *Xanthomonas* and *Stenotrophomonas*, establishing the initial understanding of the regulatory function of this family of proteins in bacteria (Qian et al. 2013; McCarthy et al. 2011). However, the role of Ax21 family proteins in non-pathogenic bacteria had not been established until this study. Here, we show that Ax21-family proteins (Lsp) in the biological control agent *L. enzymogenes* play a vital role in regulating the biosynthesis of antimicrobial secondary metabolites. This function has not been reported in any other bacterial species in which Ax21 family proteins are known to be produced.

The Lsp proteins regulate the production of HSAF and WAP-8294A2 in *L. enzymogenes*. The ability to produce antibiotics to inhibit competitors or to inactivate organisms that serve as potential nutrient sources is a critical factor for the existence of a soil-inhabiting, non-pathogenic organism, just as virulence against a host is essential for the existence of a pathogenic organism factor. The fact the Ax21 family protein are associated with competitive ability in a non-pathogen and with virulence in pathogenic bacterial species suggest that these proteins evolved to regulate key survival/ecological traits. The Lsp proteins do regulate the trait (HSAF and WAP-8294A2) critical to the ecological functioning of *L. enzymogenes*, but the deletion of *lsp* genes together and in combination had no detectable effects on the ability of the bacterium to produce lytic enzymes or to engage in twitching motility. The latter finding was particularly surprising considering that Ax21 family proteins have been shown to regulate flagella-driven swimming motility in *Xoo* and *S. maltophilia* (McCarthy et al. 2011; Ferrer-Navarro et al. 2013; Park et al. 2014). Our findings do suggest that the regulatory role of Ax21 family proteins in cell motility is variable among different members of the Xanthomonadaceae family.

In the present study, we found that the regulation of antibiotic production in *L. enzymogenes* involved three unique Lsp proteins whose genes occur in separate locations in *L. enzymogenes* genome. In contrast, only one Ax21-family protein has been reported in *Xoo* (Bahar et al. 2014), while two Ax21-like proteins (Smlt0387 and Smlt0184) were identified in *S. maltophilia* (Devos et al. 2015). Among the two Ax21-family proteins found in *S. maltophilia,* only one (Smlt0387) has been shown to function in the regulation of motility, biofilm formation and virulence (Ferrer-Navarro et al. 2013; McCarthy et al. 2011), while the role of Smlt0184 remains unknown. These studies collectively show that different members of the Xanthomonadaceae family have the potential to express different numbers of Ax21 family proteins. The biological significance for *L. enzymogenes* needing to produce three different Lsp proteins is an open question. It is also puzzling that the Lsp proteins did not exhibit an additive effect in regulating antibiotic production given that Ax21-family proteins are presumed to act as quorum molecules; indeed, relative virulence among strains of *S. maltophilia* was correlated with the amounts of Ax21 protein produced (Ferrer-Navarro et al. 2013). If the Lsp proteins are quorum sensing molecules, then a possible explanation for the non-additive phenomenon is that the three Lsp proteins are active in a trimer-like complex that requires all three proteins. It might also be possible that Lsp proteins function in a mechanism distinct from quorum sensing and that each protein acts on different successive steps in a single pathway, such that when any one step is blocked, then the entire pathway is blocked accordingly.

## Additional file

**Additional file 1: Table S1.** Confirmation of single mutation by PCR in this study. **Table S2.** Validation of double or triple mutation by PCR in this study. **Figure S1.** Lsp proteins contributed to the growth pattern of *Lysobacter enzymogenes*. **Figure S2.** Genomic organization of three Lsp-coding genes in *Lysobacter enzymogenes*.

## Abbreviations

HSAF: heat stable antifungal factor
Lsp: small molecule protein in *Lysobacter enzymogenes*
*Xoo*: *Xanthomonas oryzae* pv. *oryzae*
Omp1X: outer membrane protein 1 in *Xoo*
*Xoc*: *Xanthomonas oryzae* pv. *oryzicola*
MRSA: methicillin-resistant *Staphylococcus aureus*
T4P: type IV pili
Km: kanamycin
Gm: gentamicin
PCR: polymerase chain reaction
CMC: carboxymethyl cellulose
HPLC: high-performance liquid chromatography
TFA: trifluoroacetic acid
