# Supplementary material for: Lsp family proteins regulate antibiotic biosynthesis in Lysobacter enzymogenes OH11
Source: AMB Express. 2017 Jun 13;7:123. doi: 10.1186/s13568-017-0421-2 (PMC5469723; doi:10.1186/s13568-017-0421-2)
Supplement: Supplementary file 1 — Additional file 1: Table S1. Confirmation of single mutation by PCR in this study. Table S2. Validation of double or triple mutation by PCR in this study. Figure S1. Lsp proteins contributed to the growth pattern of Lysobacter enzymogenes. Figure S2. Genomic organization of three Lsp-coding genes in Lysobacter enzymogenes. [file 13568_2017_421_MOESM1_ESM.docx]

**Supplementary materials**

**Lsp family proteins regulate antibiotic biosynthesis in *Lysobacter enzymogenes* OH11**

**Ruping Wang^1, 2^, Huiyong Xu^1^, Yangyang Zhao^1^, Juan Zhang^2^, Gary Y Yuen^3^, Guoliang Qian^2^, and Fengquan Liu^1,2*^**

^1^Institute of Plant Protection, Jiangsu Academy of Agricultural Sciences, Nanjing 210014, China

^2^College of Plant Protection, Nanjing Agricultural University, Nanjing 210095, China/Key Laboratory of Integrated Management of Crop Diseases and Pests (Nanjing Agricultural University), Ministry of Education

^3^Department of Plant Pathology, University of Nebraska-Lincoln, Lincoln, Nebraska 68588, United States

**Email addresses**

Ruping Wang: [2013102043@njau.edu.cn](mailto:2013102043@njau.edu.cn)

Huiyong Xu: [xuhy0921@163.com](mailto:xuhy0921@163.com)

Yangyang Zhao: yyzhao2016@163.com

Juan Zhang : [2011102027@njau.edu.cn](mailto:2011102027@njau.edu.cn)

Gary Y Yuen : [gyuen1@unl.edu](mailto:gyuen1@unl.edu)

Guoliang Qian：[glqian@njau.edu.cn](mailto:glqian@njau.edu.cn)

Fengquan Liu*：[fqliu20011@sina.com](mailto:fqliu20011@sina.com) (* corresponding author)

Postal address: No. 50 Zhongling Street, Jiangsu Academy of Agricultural Sciences, Nanjing City, Jiangsu Province, 210014; Tel & Fax: +86-25-84390277.

**Table S1 Confirmation of single mutation by PCR in this study**

| Mutant | Selected  primers^a^ | Expected size from wild-type OH11 | Expected size from deletion mutant | PCR confirmation^b^ |
| --- | --- | --- | --- | --- |
| Δ*lsp1* | *lsp 1*-F1/R2 | 2254 bp | 1710 bp |  |
| Δ*lsp2* | *lsp2*- F1/R2 | 2178 bp | 1663 bp |  |
| Δ*lsp-3* | *lsp3*- F1/R2 | 1376 bp | 932 bp | 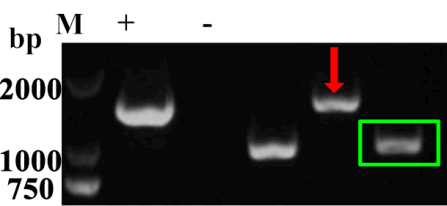 |

^a^ Primers sequence used here were provided in the above Table 2.

^b^ Red arrow and green box corresponds the expected size with the same color on the left part, respectively.

**Table S2 Validation of double or triple mutation by PCR in this study**

| Target mutant | Selected  primers^a^ | Expected size /parent mutant^b^ | Expected size from target mutant | PCR confirmation^c^ |
| --- | --- | --- | --- | --- |
| Δ*lsp12* | *lsp2*-F1/R2 | 2178 bp/  Δ*lsp1* | 1663 bp | 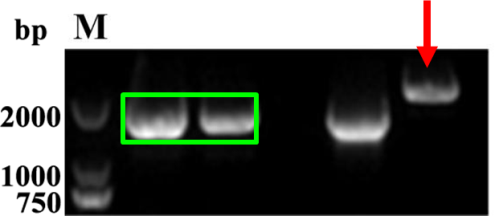 |
| Δ*lsp23* | *lsp2*-F1/R2 | 2178 bp/  Δ*lsp3* | 1663 bp | 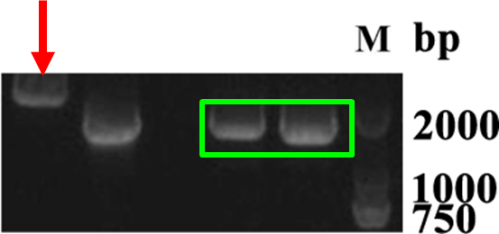 |
| Δ*lsp13* | *lsp3*-F1/R2 | 1376 bp/  Δ*lsp1* | 932 bp | 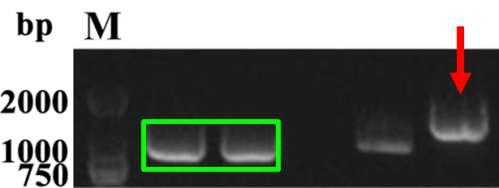 |
| Δ*lsp123* | *lsp2*-F1/R2 | 2178 bp/  Δ*lsp13* | 1663 bp | 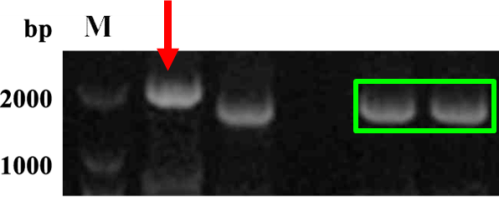 |

^a^ Primers sequence used here were provided in the above Table 2.

^b^ The mutant underlining was the parent mutant, which was used for generation of the double or triple mutation.

^c^ Red arrow and green box corresponds the expected size with the same color on the left part, respectively.


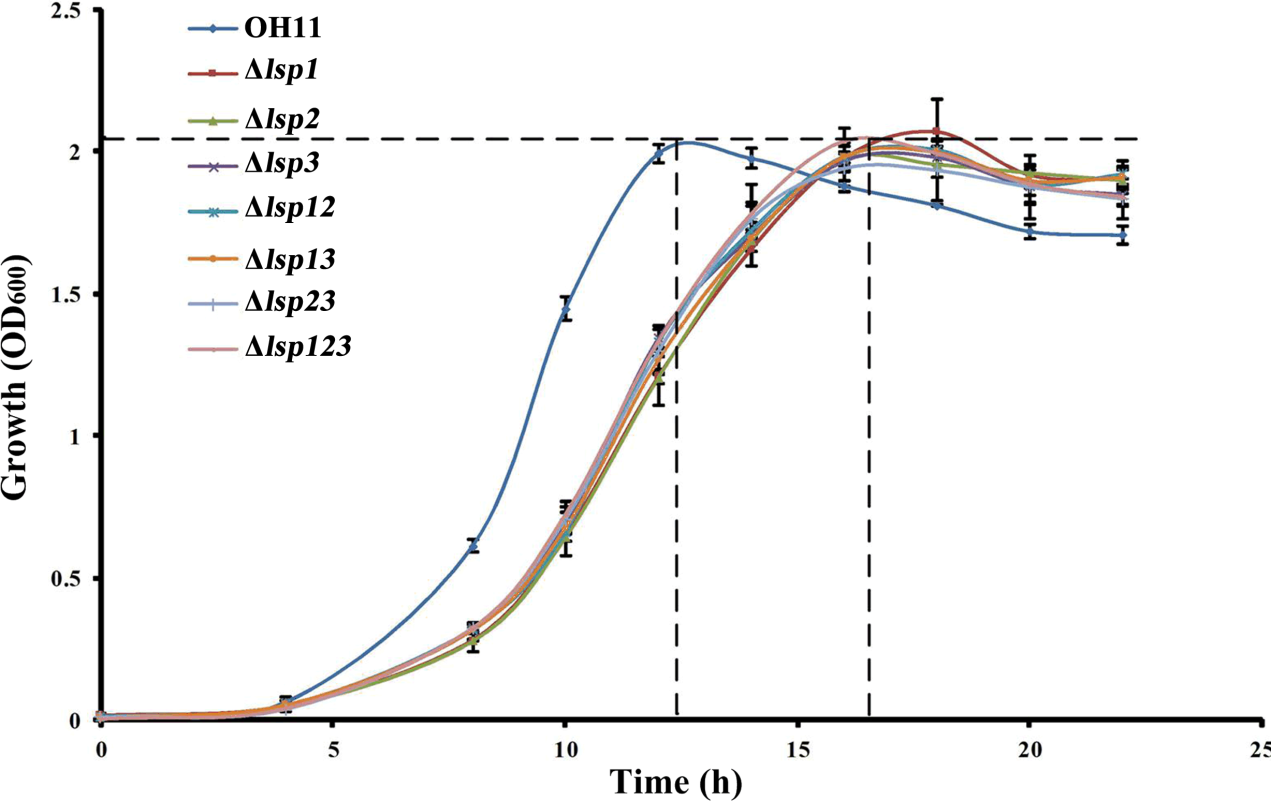


**Figure S1. Lsp proteins contributed to the growth pattern of *Lysobacter enzymogenes*.** All test *lsp* mutants exhibited an altered growth pattern that was taken place in the logarithmic phase and originated the delay in reaching the similar stationary phase compared with that of the wild-type OH11. OH11 = wild-type strain of *L. enzymogenes*; Δ*lsp1*, Δ*lsp2* and Δ*lsp3* = in-frame deletion mutants of *lsp1*, *lsp2* and *lsp3*, respectively; Δ*lsp12*, Δ*lsp13* and Δ*lsp23* = double in-frame deletion mutants of *lsp1* and *lsp2*, *lsp1* and *lsp3* and *lsp2* and *lsp3*, respectively; Δ*lsp123* = the triple deletion mutant lacking *lsp1*, *lsp2* and *lsp3*.


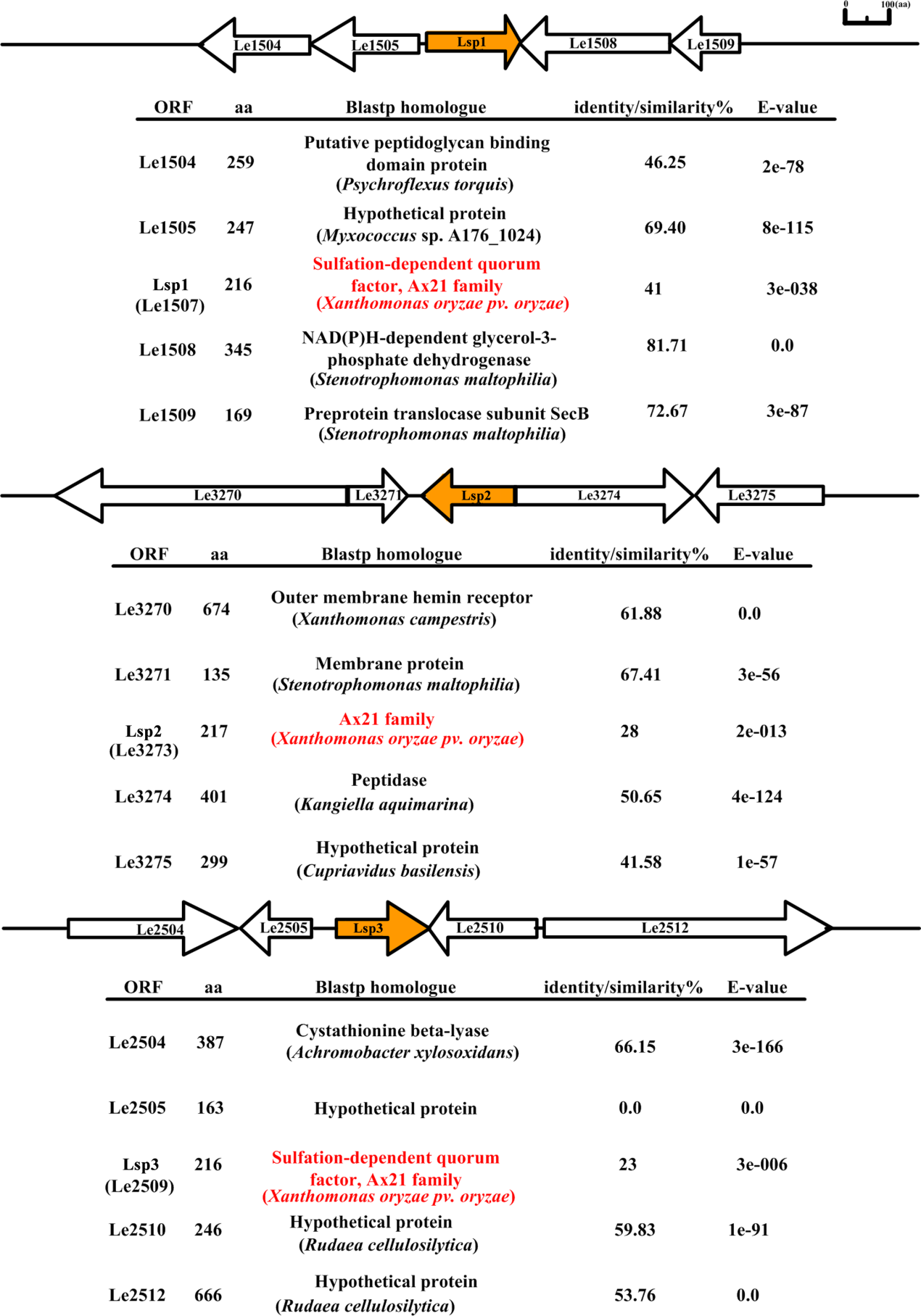


**Figure S2. Genomic organization of three Lsp-coding genes in *Lysobacter enzymogenes*.** Each gene and its coding direction is highlighted in yellow arrow; information regarding each gene is presented in red. Information regarding neighboring genes is also provided in this figure.
